# Corncob-Derived Xylo-Oligosaccharides Attenuate Acrylamide-Induced Gut–Liver Toxicity in Mice, Associated with Modulation of Gut Microbiota and SCFA Metabolism

**DOI:** 10.3390/biology15191684

**Published:** 2026-09-22

**Authors:** Le He, Yanjuan Chen, Ruiping Luo, Zhongke Sun, Erting Liu, Lili Li, Zonghao Yue

**Affiliations:** 1 School of Life Sciences and Agronomy, Zhoukou Normal University, Zhoukou 466001, China; 20122040@zknu.edu.cn (L.H.); ruipingluo@zknu.edu.cn (R.L.); 2 School of Mechanical and Electrical Engineering, Zhoukou Normal University, Zhoukou 466001, China; 20172033@zknu.edu.cn; 3 School of Biological Engineering, Henan University of Technology, Zhengzhou 450001, China; sun_microbiol@sina.com; 4 Henan Heagreen Bio-Technology Co., Ltd., Zhoukou 466001, China; ertin2026@163.com

**Keywords:** xylo-oligosaccharides, acrylamide, mice, gut–liver toxicity, gut microbiota, short chain fatty acid

## Abstract

Acrylamide (AA) is a common foodborne toxicant formed during the thermal processing of starch-rich foods and poses significant risks to the gut–liver axis. Although corncob-derived xylo-oligosaccharides (XOS) have emerged as promising prebiotics with multiple bioactivities, their protective effects against foodborne toxicants have not been explored. This study investigated the ameliorative effects of dietary corncob-derived XOS on AA-induced gut–liver toxicity and the underlying mechanisms in mice. Female C57BL/6J mice were gavaged with AA and concurrently fed diets with or without 7.0% XOS for 7 weeks. The results showed that XOS effectively alleviated AA-induced gut–liver toxicity, as evidenced by attenuated intestinal and hepatic inflammation (decreased pro-inflammatory cytokines IL-6 in colon, TNF-α, and IL1-β in liver; increased anti-inflammatory IL-10 in colon and liver), restored intestinal barrier integrity (upregulated *Zo-1*, *Occludin*, and *Claudin-1* mRNA, along with increased goblet cell numbers and mucus secretion), and ameliorated hepatic oxidative stress (upregulated *Nrf2*/*Nqo1*/*Ho1* mRNA expression, restored CAT activity, and reduced H_2_O_2_ accumulation). 16S sequencing revealed that XOS supplementation significantly increased the Shannon index in AA-exposed mice compared with the AA group, suggesting enhanced cecal microbial diversity, although AA exposure did not significantly reduce α-diversity. Further analysis showed that XOS reshaped the community composition, particularly by reducing pro-inflammatory genera (*Dubosiella*, *Desulfovibrio*, *Faecalibaculum*, and *Faecalimonas*) and enriching short chain fatty acid (SCFA)-producing bacteria (*Bifidobacterium*, *norank_f__Muribaculaceae*, and *Akkermansia*). Furthermore, XOS significantly elevated SCFA levels in AA-treated mice, including acetic, propionic, butyric, valeric, and isovaleric acids. Collectively, these findings demonstrate that dietary XOS ameliorates AA-induced gut–liver toxicity in mice when co-administered with AA. This protective effect was associated with modulation of the gut microbiota and SCFA metabolism, providing a basis for future studies exploring the potential of XOS against foodborne contaminant toxicity.

## Simple Summary

Acrylamide (AA), a prevalent foodborne contaminant formed during high-temperature food processing, poses significant health risks; however, effective and practical dietary countermeasures remain scarce. Our study provides compelling evidence that a prebiotic, xylo-oligosaccharides (XOS) derived from corncobs, effectively mitigates AA-induced gut–liver toxicity in mice, characterized by alleviation of colonic and hepatic inflammation, amelioration of intestinal barrier dysfunction, and reduction in hepatic oxidative stress. These protective effects were closely associated with the remodeling of gut microbiota, characterized by a marked enrichment of short-chain fatty acid (SCFA)-producing genera and a concomitant reduction in pro-inflammatory taxa, resulting in significantly elevated SCFA levels. These findings indicate that corncob-derived XOS represents a potential prebiotic candidate for counteracting AA-induced toxicity and support further investigation into its value for future applications in the food safety field.

## 1. Introduction

Acrylamide (AA) is a hazardous contaminant generated during the thermal processing of food. Numerous studies have confirmed its widespread presence in various food items, particularly potato products, cereal products, coffee, and other starch-rich foods, with reported levels reaching up to 3151.8 μg/kg [1,2,3,4]. Toxicological studies have shown that AA exhibits carcinogenic, neurotoxic, cardiotoxic, and hepatotoxic effects [5]. Previous work from our laboratory revealed that AA induced glucose metabolism disorders, gut microbiota dysbiosis, and intestinal damage [6,7]. Recent epidemiological evidence has linked dietary AA exposure to an increased risk of several diseases, including type 2 diabetes, cardiovascular disease, anxiety, and depression [8,9,10]. Consequently, mitigating the long-term public health risks posed by dietary AA has drawn significant global regulatory and research attention.

Although completely preventing the formation of AA at the source is an ideal approach, no technology is currently available to achieve this goal. Current techniques for reducing AA levels, including raw material selection, process optimization, and food additive application, face inherent limitations, such as inconsistent efficacy, adverse effects on sensory quality, narrow food-specific applicability, and potential formation of other contaminants [11,12,13,14,15]. An alternative strategy is the post-ingestion intervention with exogenous bioactive substances. Recent studies have shown that polyphenols, terpenoids, alkaloids, quinones, allicin, and polysaccharides can mitigate AA-induced toxicity [16,17,18,19]. Nevertheless, most of these bioactive compounds originate from high-value sources such as medicinal plants, edible crops, or fungi, which constrains their feasibility for large-scale public health applications.

In this context, xylo-oligosaccharides (XOS), a class of functional oligosaccharides composed of 2–7 xylose units linked by β-1,4-glycosidic bonds, have attracted attention [20]. XOS can be efficiently extracted from agricultural residues, particularly from corncobs, a major by-product of corn processing with an estimated annual availability of 90 million tons in China alone. As an emerging prebiotic, XOS exert multiple health benefits, including gut microbiota modulation, short-chain fatty acid (SCFA) stimulation, anti-inflammatory and antioxidant activities, and intestinal barrier protection [21,22]. Notably, XOS may achieve efficacy at a lower dose than other prebiotics such as fructooligosaccharides (FOS) and galactooligosaccharides (GOS) [23]. Although the prebiotic activity of XOS has been widely acknowledged, direct experimental evidence on whether XOS can alleviate the toxicity of food contaminants, including AA, remains limited. A previous study reported that a combination of FOS and XOS attenuated AA-induced neurotoxicity in maternal and fetal rats [24]. However, the protective effects of XOS alone against AA-induced gut–liver toxicity have not been explored.

Therefore, this study investigated the protective effects of corncob-derived XOS against AA-induced gut–liver toxicity in C57BL/6J mice and explored the underlying mechanisms using a combination of techniques, including 16S rRNA gene amplicon sequencing, histopathological examination, targeted metabolomics for SCFAs, quantitative real-time PCR (qPCR), and biochemical assays. Our findings highlight the potential of XOS as a prebiotic candidate for further investigation in the context of foodborne contaminant toxicity.

## 2. Materials and Methods

### 2.1. Materials and Reagents

Acrylamide (AA, purity ≥ 99.9%) was purchased from Aladdin (Shanghai, China). Corncob-derived XOS powder (purity ≥ 95%) was generously provided by Henan Heagreen Bio-technology Co., Ltd. (Zhoukou, China). The physicochemical properties of XOS are provided in Table 1. The AIN-93M diet with or without 7.0% XOS (*w*/*w*) was obtained from Research Diets (New Brunswick, NJ, USA). Diet composition is detailed in Supplementary Table S1. The colorimetric kits for aspartate aminotransferase (AST), alanine aminotransferase (ALT), superoxide dismutase (SOD), catalase (CAT), hydrogen peroxide (H_2_O_2_), and glutathione (GSH) were acquired from Boxbio (Beijing, China). The ELISA kits for tumor necrosis factor α (TNF-α), interleukin-1β (IL1-β), interleukin-6 (IL-6), and interleukin-10 (IL-10) were purchased from Elabscience (Wuhan, China). RNA extraction and qPCR kits, including FreeZol Reagent, HiScript II Q RT SuperMix, and ChamQ SYBR qPCR Master Mix, were purchased from Vazyme (Nanjing, China).

### 2.2. Animal Experimental Design

Twenty-four female SPF C57BL/6J mice (Six-week-old, Charles River Laboratories, Beijing, China) were acclimated for one week and then randomized into four groups using a random number table method (n = 6/group, 3/cage): control, AA treatment, XOS treatment, and XOS+AA treatment. The reason for selecting females is based on a previous report which indicates that females may be more prone to absorbing AA than males [25]. Mice in the control and AA groups were fed an AIN-93M diet, whereas those in the XOS and XOS+AA groups received an AIN-93M diet containing 7.0% XOS. In addition, mice in the AA and XOS+AA groups received 15 mg/kg·bw AA by oral gavage once daily in a volume of 6 mL/kg body weight, whereas those in the control and XOS groups received an equal volume of deionized water. During the experiment, mice were housed at 25 ± 1 °C, 50 ± 5% humidity, and a 12 h light/dark cycle. Cage positions were rotated weekly. Body weight, food intake, and water consumption were recorded weekly. After 7 weeks of treatment, the mice were fasted for 8 h and anesthetized with 1% sodium pentobarbital at a dose of 50 mg/kg·bw via intraperitoneal injection. Then, mice were euthanized by cervical dislocation. Serum, cecal contents, colonic contents, colonic tissue, and liver were collected as described in our previous study [26].

All animal procedures were approved by the Biomedical Ethics Committee of Zhoukou Normal University (approval number: ZKNU-20260026; approved on 21 March 2026), and performed in accordance with the ARRIVE 2.0 guidelines. Due to the nature of the dietary intervention, the investigators responsible for animal care and treatment administration were aware of group assignments. However, all outcome assessments, including histopathological evaluation, biochemical assays, and data analysis, were performed by investigators blinded to group allocation by means of coded sample labels.

### 2.3. Histopathological Analysis

Liver and colonic samples were fixed in 4% paraformaldehyde, ethanol-dehydrated, xylene-cleared, paraffin-embedded, and sectioned at 5 μm. Thereafter, the sections were stained with hematoxylin and eosin (H&E) or Alcian blue and periodic acid-Schiff (AB-PAS). Histopathological images were obtained using a slide scanner (3DHISTECH, Budapest, Hungary). Quantification was performed using Image-Pro Plus 6.0 software, and one AB-PAS-stained section per mouse was analyzed. At 50× magnification, the AB-PAS-positive area and the corresponding colonic mucosal layer area were measured in each AB-PAS-stained section, and the percentage of positive area was calculated as (positive area/mucosal layer area) × 100. The AB-PAS-positive area was defined using the default color threshold settings, applied uniformly to all images. Three intact crypts per section were selected; the crypt epithelial length was measured along the crypt axis using Image-Pro Plus 6.0, and the number of goblet cells within each crypt was counted. The number of goblet cells per unit epithelial length was calculated as goblet cell number/crypt epithelial length. During the experiment, all reagents were obtained from Servicebio (Wuhan, China), except for ethanol and xylene, which were obtained from Sinopharm Chemical Reagent (Shanghai, China).

### 2.4. Biochemical Analysis

Serum AST and ALT activities, hepatic SOD and CAT activities, and hepatic H_2_O_2_ and GSH levels were measured using colorimetric kits. Liver and colonic levels of TNF-α, IL-1β, IL-6, and IL-10 were determined using ELISA kits. All assays were performed according to the manufacturers’ protocols.

### 2.5. RNA Extraction and qPCR Assay

Total RNA was isolated from liver and colonic samples with FreeZol Reagent and quantified using a NanoDrop Lite Plus (Thermo Fisher, Waltham, MA, USA). Subsequently, RNA (1.0 μg) was reverse-transcribed into cDNA using the HiScript II Q RT SuperMix, and qPCR was performed on a CFX96 system (Bio-Rad, Hercules, CA, USA) using the ChamQ SYBR qPCR Master Mix. Primer sequences are provided in Supplementary Table S2. Target gene expression was calculated by the 2^−ΔΔCt^ method and normalized to *β-actin*.

### 2.6. 16S rRNA Amplicon Sequencing

Total DNA was extracted from cecal contents using a stool DNA isolation kit (Yuhua, Shanghai, China). The V3–V4 region of 16S rRNA was amplified with primers 338F/806R. After purification and quantification, the PCR products were used to construct sequencing libraries with the NEXTFLEX Rapid DNA-Seq Kit (Bio Scientific, Austin, TX, USA). The libraries were sequenced on an Illumina NextSeq 2000 system (Illumina, San Diego, CA, USA) by Majorbio (Shanghai, China). Bioinformatics analyses, including α-diversity, β-diversity, and taxonomic composition, were performed using the Majorbio Cloud Platform (https://cloud.majorbio.com, accessed on 30 April 2026). Detailed analysis procedures are provided in the Supplementary Methods. Differential abundance analysis was performed using one-way ANOVA, followed by a Tukey test with FDR-adjusted *p*-value < 0.05. The sample-to-group mapping and cage information for the 16S rRNA sequencing samples are provided in Supplementary Table S3.

### 2.7. Determination of SCFAs

Colonic contents (50 mg) were mixed with 100 μL 20% H_3_PO_4_ and 1.0 mL isopropyl ether containing 500 μM 4-methylvaleric acid. After vortexing, the mixtures were centrifuged for 20 min at 4 °C and 13,000× *g*. The supernatants were then analyzed for SCFA contents by gas chromatography–mass spectrometry (GC-MS) using an DB-FFAP GC system coupled to a 5977B MSD mass spectrometer (Agilent, Santa Clara, CA, USA). Detailed GC and MS parameters are provided in the Supplementary Methods. After detection, peak areas and retention times were extracted using MSD ChemStation software, version E.02.02 SP2 and SCFA contents were calculated from standard curves.

### 2.8. Statistical Analysis

All animals were included in the analysis. Data are presented as mean ± SD. Group comparisons were performed using one-way ANOVA followed by Tukey or Tamhane’s T2 test (based on variance equality) with SPSS 19.0 (IBM, Armonk, NY, USA). Statistical significance was set at *p* < 0.05.

## 3. Results

### 3.1. XOS Ameliorates AA-Induced Colonic Inflammation in Mice

In this study, the body weights of the mice were significantly lower in the AA-exposed group than in the control group (Figure 1B). Although not statistically significant, XOS supplementation increased the body weight of AA-exposed mice (Figure 1B). H&E staining and inflammatory cytokine measurements revealed that AA exposure induced colonic inflammatory cell infiltration and elevated levels of the pro-inflammatory cytokine IL6 (Figure 1C,F). In contrast, XOS supplementation significantly ameliorated colonic inflammation, decreased IL-6 levels, and increased levels of the anti-inflammatory cytokine IL-10 in AA-exposed mice compared with the AA group (Figure 1C,F,G).

### 3.2. XOS Improves AA-Induced Colonic Barrier Dysfunction in Mice

AB-PAS staining showed that AA exposure disrupted the colonic mucus barrier, as evidenced by a significant reduction in the number of goblet cells and AB-PAS-positive area (Figure 2A–C). In contrast, XOS supplementation restored both parameters in AA-treated mice to levels comparable to those in the control group. In addition, AA exposure significantly downregulated the mRNA levels of the colonic tight junction (TJ) genes zonula occludens-1 (*Zo-1*), *Occludin*, *Claudin-1*, and *Claudin-5* compared with the control group. However, XOS treatment markedly increased the mRNA levels of these TJ genes in AA-exposed mice, except for *Claudin-5*, which was not significantly restored by XOS (Figure 2D–G).

### 3.3. XOS Ameliorates AA-Induced Liver Inflammation in Mice

Compared with the control group, AA-exposed mice exhibited marked inflammatory cell infiltration in the liver, significantly elevated serum AST levels, and increased hepatic levels of TNF-α and IL1-β (Figure 3A,B,D,E). In contrast, XOS supplementation reversed these changes and significantly increased IL-10 levels (Figure 3A,B,D,E,G).

### 3.4. XOS Ameliorates AA-Induced Oxidative Stress in Liver of Mice

AA exposure significantly decreased CAT activity, downregulated the mRNA levels of nuclear factor erythroid 2-related factor 2 (*Nrf2*), NADPH:quinone oxidoreductase 1 (*Nqo1*), and heme oxygenase-1 (*Ho1*), and increased H_2_O_2_ and GSH levels in the liver compared with the control group (Figure 4B–G). However, XOS supplementation significantly increased CAT activity, elevated mRNA levels of *Nrf2*, *Nqo1*, and *Ho1*, and decreased H_2_O_2_ levels in AA-exposed mice compared with those in the AA group (Figure 4B,C,E–G). In contrast, XOS supplementation did not significantly alter hepatic GSH levels compared with the AA group, although GSH levels in the AA+XOS group remained significantly higher than those in the control group (Figure 4D).

### 3.5. XOS Alters Gut Microbial Diversity and Community Composition in AA-Exposed Mice

16S rRNA sequencing of cecal contents showed that AA exposure decreased the α-diversity indices (Chao and Shannon), although the decrease was not statistically significant (Figure 5A,B). However, XOS supplementation significantly increased the Shannon index in mice exposed to AA (Figure 5B). Principal coordinate analysis (PCoA) demonstrated a clear separation of samples among the different treatment groups (Figure 5C). ANOSIM further confirmed that the differences in gut microbial community structure among groups were significant (R = 0.94, *p* = 0.001). Microbial composition analysis showed that *Lactobacillus* and *Desulfovibrio* were the most abundant genera in the control and AA-treated groups, whereas *Bifidobacterium* and *Allobaculum* were the most abundant genera in the XOS and XOS+AA groups (Figure 5D; Table S4). Differential abundance analysis at the genus level (relative abundance ≥ 1.0%) indicated that AA exposure significantly decreased the abundance of *Blautia* and *Candidatus_Saccharimonas* and significantly increased the abundance of *Dubosiella* compared with the control group (Figure 5E; Table S5). However, XOS supplementation significantly decreased the abundance of *Dubosiella*, *Lactobacillus*, *Desulfovibrio*, *Faecalibaculum*, and *Faecalimonas* and significantly increased the abundance of *Bifidobacterium*, *norank_f__Muribaculaceae*, and *Akkermansia* in AA-exposed mice (Figure 5E; Table S5).

### 3.6. XOS Increases SCFA Contents in Colonic Contents in AA-Exposed Mice

As shown in Figure 6, AA exposure did not significantly alter SCFA contents in the colonic contents compared with the control group, although a decreasing trend was observed. However, in AA-exposed mice, XOS supplementation significantly increased total SCFA content to 2.99 times the level in the AA group, with the acetic, propionic, butyric, valeric, and isovaleric acid contents being 3.50, 1.58, 2.75, 2.96, and 2.05 times the levels in the AA group, respectively.

### 3.7. Correlation Analysis Among Differential Genera, SCFAs, Intestinal–Liver Parameters

Spearman’s correlation analysis revealed that the abundances of *Bifidobacterium*, *norank_f__Muribaculaceae*, and *Akkermansia* were positively correlated with SCFA contents (Figure 7A). In contrast, the abundances of *Lactobacillus*, *Dubosiella*, *Desulfovibrio*, *Faecalibaculum*, *Faecalimonas*, and *Blautia* were negatively correlated with SCFA contents (Figure 7A). Moreover, SCFA contents were positively correlated with the mRNA expression of colonic TJ genes, mucus barrier-related parameters, and colonic IL-10 level, but negatively correlated with colonic IL-6 level (Figure 7B). Furthermore, SCFA contents were positively correlated with hepatic IL-10 level, CAT activity, and the mRNA expression of *Nrf2*, *Ho1*, and *Nqo1*, but negatively correlated with hepatic TNF-α and H_2_O_2_ levels (Figure 7B).

## 4. Discussion

AA is a ubiquitous food-borne toxicant. In the present study, the 15 mg/kg bw/day AA dose was selected based on two considerations. First, previous studies have shown that 15 mg/kg bw/day AA induces intestinal and hepatic injury in rodents [27,28,29]. Second, the most sensitive non-neoplastic endpoint for acrylamide in rodents is peripheral neurotoxicity. For axonal degeneration in the sciatic nerve, a NOAEL of 1.84 mg/kg bw/day and a LOAEL of 4.02 mg/kg bw/day have been reported in female F344 rats [30,31]. Using body surface area normalization (Km rat = 6; Km human = 37) from Reagan-Shaw et al. [32], this LOAEL corresponds to a human equivalent dose (HED) of approximately 0.65 mg/kg bw/day. The 15 mg/kg bw/day dose used in mice corresponds to an HED of approximately 1.2 mg/kg bw/day, which is approximately 1.8-fold higher than the HED for the neurotoxicity LOAEL. Therefore, this dose is within the high-dose range. Our results showed that AA exposure led to colonic inflammatory infiltration and the accumulation of the pro-inflammatory cytokine IL-6, consistent with previous reports demonstrating AA-induced intestinal inflammation [26,33]. XOS is a prebiotic that can be derived from corncob and has been reported to confer multiple intestinal benefits [21]. Dietary XOS at 7.0% has been shown to significantly modulate gut microbial composition and increase cecal SCFA levels in mice [34,35]. In this study, 7.0% XOS (the human-equivalent dose is approximately 34 g/day) was supplemented to AA-exposed mice, and we found that XOS supplementation significantly alleviated AA-induced colonic inflammatory responses and promoted the accumulation of the anti-inflammatory cytokine IL-10, suggesting that XOS effectively ameliorated colonic inflammation in AA-exposed mice.

Intestinal inflammation typically compromises intestinal barrier integrity [36]. The intestinal barrier is a multilayered system in which the mucus layer, formed by mucins secreted by goblet cells, constitutes the first chemical barrier, while the epithelial cells connected by tight junction (TJ) proteins form the critical physical barrier [37]. An intact intestinal barrier effectively prevents the invasion of pathogens and toxins and is essential for maintaining intestinal health [38]. In this study, AA exposure reduced mucus secretion and goblet cell numbers, accompanied by significantly downregulated mRNA expression of the TJ genes *Zo-1*, *Occludin*, *Claudin-1*, and *Claudin-5*, indicating that AA disrupted both the mucus layer (chemical barrier) and the TJ-mediated physical barrier. Similar results have been reported in previous studies [33]. Importantly, XOS supplementation reversed these AA-induced changes, suggesting that XOS may repair AA-induced intestinal barrier dysfunction by simultaneously enhancing physical and chemical barriers.

In addition to intestinal damage, AA has also been reported to induce hepatotoxicity [39,40]. In line with these reports, our study confirmed AA-induced liver injury, as evidenced by elevated hepatic inflammatory infiltration and pro-inflammatory cytokine levels. Serum AST activity was also elevated in AA-exposed mice; however, serum ALT activity did not differ significantly among groups. Such inconsistent changes in AST and ALT have been reported in a previous AA toxicology study, which showed that AA-treated rats exhibited increased serum AST activity but decreased ALT activity [41]. In addition, an in vitro study has shown that AST leakage from hepatocytes occurs earlier and is more sensitive than ALT leakage under AA exposure [42]. Given that AST is not liver-specific, the elevated serum AST activity may originate from extrahepatic tissues, and further studies are needed to clarify its tissue source. In contrast, XOS supplementation effectively reversed these AA-induced biochemical and histopathological changes, indicating that XOS alleviated hepatotoxicity in mice exposed to AA. Research evidence has shown that AA-induced hepatotoxicity is closely associated with oxidative stress [43]. In the present study, AA exposure significantly decreased CAT activity and elevated H_2_O_2_ levels, confirming oxidative stress in the liver. Moreover, AA exposure markedly downregulated the mRNA expression of *Nrf2*, *Nqo1*, and *Ho1*. Similarly, previous studies have shown that AA exposure significantly decreases their expression at the protein level [44,45,46]. *Nrf2* is a transcription factor that plays a critical role in combating oxidative stress. It upregulates the expression of various antioxidant enzyme genes, including *Nqo1*, *Ho1*, and *Cat*, thereby alleviating oxidative damage [47,48]. Importantly, XOS supplementation restored CAT activity, normalized H_2_O_2_ levels, and significantly upregulated the mRNA expression of *Nrf2*, *Nqo1*, and *Ho1* in mice exposed to AA. Collectively, these results suggest that increased *Nrf2* transcript abundance and upregulation of *Nrf2*-targeted antioxidant genes may contribute to the alleviation of AA-induced oxidative stress by XOS, although further studies including nuclear translocation assays are required to confirm the direct activation of *Nrf2*. In addition, we observed that GSH levels were elevated by AA exposure, but XOS supplementation did not significantly alter GSH levels in AA-exposed mice compared with the AA group. AA is an electrophile that can conjugate with GSH [49]. The elevation of GSH may reflect an adaptive increase in GSH synthesis to counteract oxidative damage.

The gut microbiota is critical for gut–liver axis homeostasis [50]. Previous studies have demonstrated that AA induces dysbiosis in the gut microbiota [6,51,52]. In the present study, XOS supplementation significantly increased the Shannon index in AA-exposed mice compared with AA group, suggesting enhanced microbial diversity. At the genus level, we observed a decreased abundance of *Candidatus_Saccharimonas* and an increased abundance of *Dubosiella* in the AA-exposed group. *Candidatus_Saccharimonas* is considered to be negatively associated with host inflammation [53,54]. *Dubosiella* is reported to proliferate in mice fed dietary advanced glycation end products and is positively associated with tissue inflammation [55]. Therefore, changes in the abundance of these two bacteria may partially account for the colonic inflammation induced by AA. In contrast, XOS supplementation significantly reduced the abundance of *Dubosiella* and three other inflammation-associated genera (*Desulfovibrio*, *Faecalibaculum*, and *Faecalimonas*). *Desulfovibrio* is commonly considered harmful and can induce metabolic diseases and inflammation [56]. *Faecalibaculum* is a pro-inflammatory genus that damages the intestinal barrier and exacerbates intestinal inflammatory disorders [57,58]. A previous study showed that *Faecalimonas* is associated with inflammatory responses in mice with colitis [59]. Therefore, the reduction in these pro-inflammatory bacteria may play a key role in XOS-mediated amelioration of colonic inflammation. In addition to inflammation-associated genera, XOS supplementation significantly increased the abundance of *Bifidobacterium*, *norank_f__Muribaculaceae*, and *Akkermansia*. Notably, these genera are known probiotics and have been confirmed to produce SCFAs [60,61,62]. Therefore, XOS-induced enrichment of these SCFA-producing bacteria likely contributed to the elevated SCFA levels observed in this study. Consistently, correlation analysis revealed a positive correlation between these SCFA-producing bacteria and SCFA contents. Furthermore, XOS supplementation decreased the abundance of *Lactobacillus*, likely due to competitive exclusion by the highly efficient XOS utilizer *Bifidobacterium*. Similar results were observed in an in vitro study using antibiotic-stressed elderly gut microbiota, in which XOS reduced *Lactobacillus* abundance [63].

SCFAs are fatty acids with 1–6 carbon atoms produced by the fermentation of undigested dietary fiber by the gut microbiota in the colon [64]. Numerous studies have shown that SCFAs can upregulate the expression of TJ proteins and mucin-related proteins, inhibit the secretion of pro-inflammatory factors, and increase the production of anti-inflammatory factors, thereby exerting anti-inflammatory and barrier-protective effects in the intestine [65,66,67]. In the present study, XOS treatment increased SCFA contents in mice exposed to AA, suggesting that XOS alleviates colonic inflammation and intestinal barrier damage, at least in part, by elevating SCFA levels. Correlation analysis further supports these results. In addition to their local effects in the gut, SCFAs can reach the liver via the portal vein and exert direct hepatoprotective effects. For instance, SCFAs can suppress pro-inflammatory gene expression and reduce inflammatory cell infiltration, thereby reducing serum AST and ALT levels and ameliorating liver injury [68]. Moreover, SCFAs have been reported to activate the *Nrf2* defense pathway, upregulating antioxidant genes, including *Nqo1*, *Ho1*, and *Cat*, which protect the liver from oxidative stress [69]. Notably, this aligns with our observation that XOS treatment, which elevated SCFA levels, also upregulated the mRNA expression of *Nrf2* and its downstream genes *Nqo1* and *Ho1* in AA-exposed mice, accompanied by restored CAT activity and reduced hepatic inflammation. Collectively, these findings suggest that the hepatoprotective effects of XOS may be mediated, at least in part, through the elevation of SCFAs and the associated upregulation of *Nrf2*-targeted antioxidant genes, a notion further supported by correlation analysis.

However, several limitations of this study should be acknowledged. First, this study primarily evaluated the protective effect of XOS against AA-induced gut–liver toxicity under co-exposure conditions, rather than its therapeutic effect on established AA-induced damage. Future studies should first establish an AA-induced injury model and then assess whether XOS can ameliorate pre-existing AA-induced damage. Second, although our results demonstrate significant associations between XOS-induced changes in gut microbiota composition and SCFA profiles and the protective effects of XOS against AA-induced gut–liver toxicity, future studies employing fecal microbiota transplantation (FMT) in germ-free or antibiotic-treated recipient mice, as well as probiotic interventions using well-characterized SCFA-producing strains (e.g., *Bifidobacterium*), are warranted to further validate the causal role of specific microbial taxa or SCFA species. Third, our findings were obtained exclusively in a murine model, and the extent to which these results can be extrapolated to humans remains unknown. Future epidemiological investigations and well-designed human intervention trials are needed to evaluate the feasibility and efficacy of XOS as a dietary strategy for mitigating foodborne contaminant exposure in real-world settings.

## 5. Conclusions

In summary, this study demonstrated for the first time that XOS alleviated AA-induced colonic inflammation, intestinal barrier dysfunction, as well as hepatic inflammation and oxidative stress. The protective effect of XOS against AA-induced gut–liver toxicity is likely associated with the restoration of gut microbiota homeostasis, particularly increased SCFA-producing bacteria and decreased pro-inflammatory taxa, as well as the enhancement of SCFA production. This study demonstrates that XOS exerts a protective effect against AA-induced toxicity and provides a research basis for its future exploration as a prebiotic candidate in food safety-related applications.

## Figures and Tables

**Figure 1:**
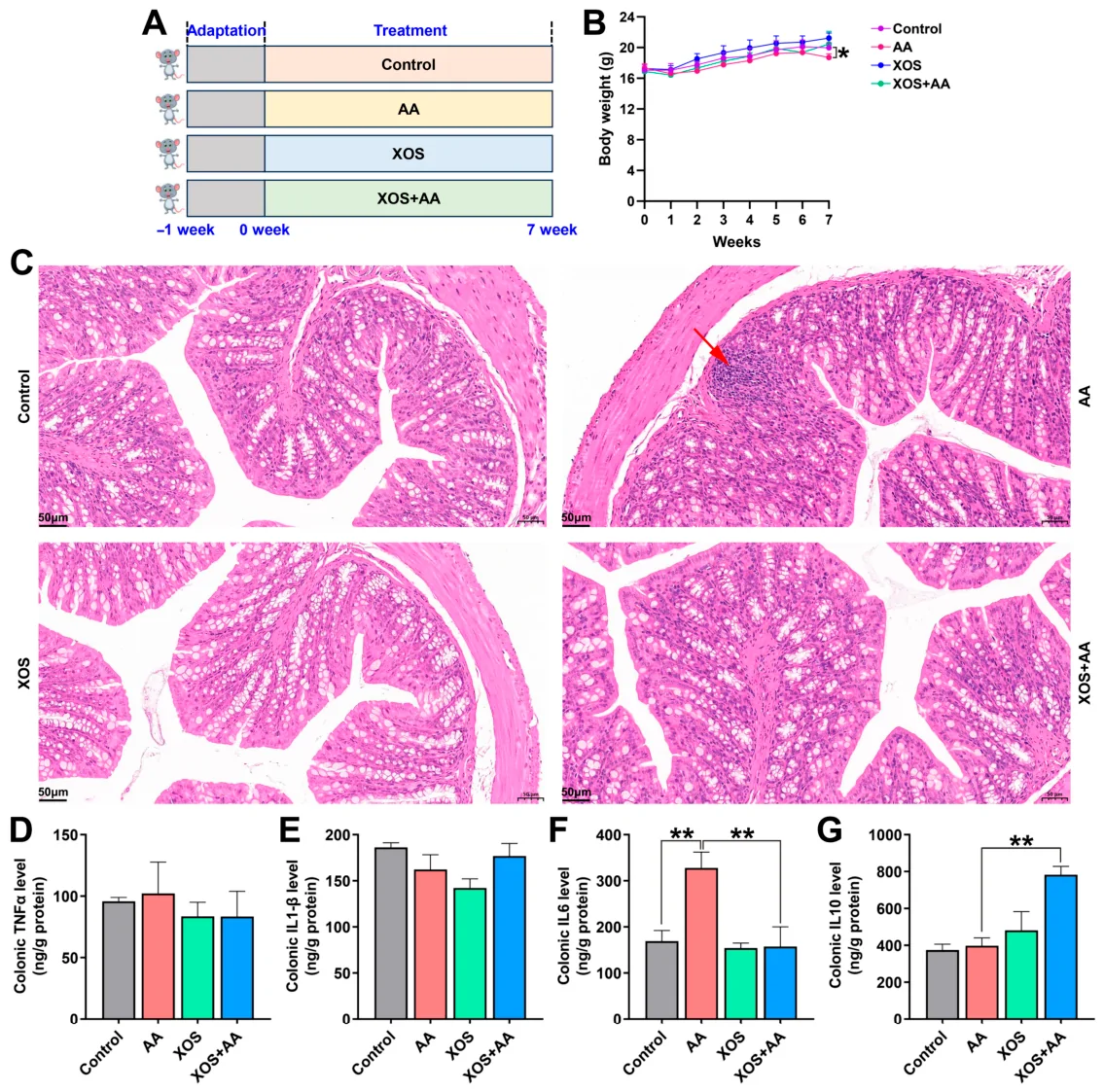
Dietary XOS mitigates AA-induced colonic inflammation in mice (n = 6/group). (**A**) Schematic of the animal experimental protocol. (**B**) Changes in the body weight of mice. (**C**) Representative H&E-stained sections of colonic tissues (scale bar: 50 μm). The red arrow indicates obvious inflammation. (**D**–**G**) Colonic levels of the inflammatory cytokines TNF-α, IL1-β, IL-6, and IL-10. * *p* < 0.05 and ** *p* < 0.01.

**Figure 2:**
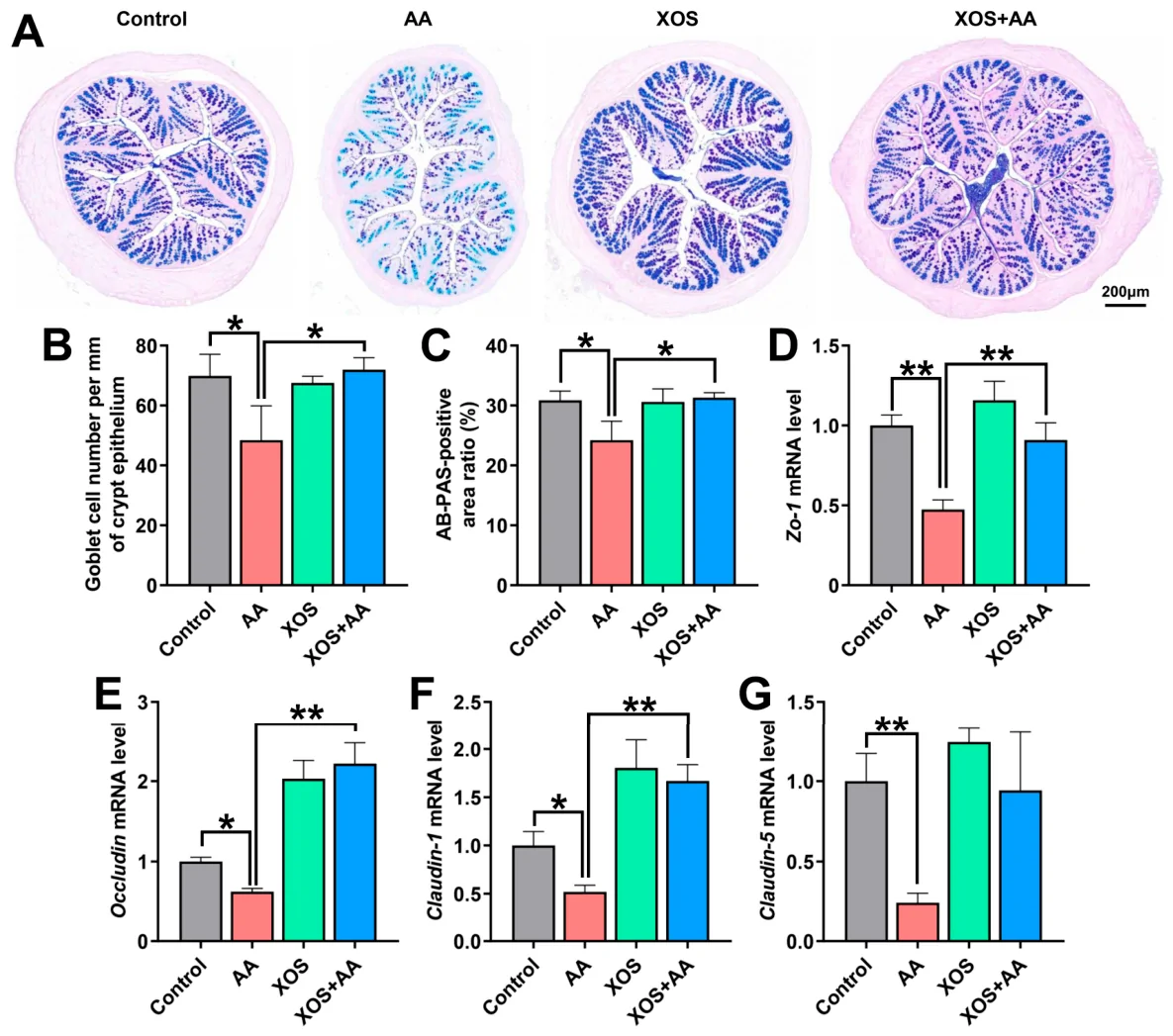
Dietary XOS ameliorates AA-induced intestinal barrier dysfunction in mice (n = 6/group). (**A**) Representative AB-PAS-stained sections of colonic tissues (scale bar: 200 μm). AB-PAS staining labels mucin-containing goblet cells: neutral mucins appear magenta, acidic mucins blue, and mixed mucins purple; staining intensity reflects relative mucin content. (**B**) Goblet cell number per mm of crypt epithelium. (**C**) Ratio of AB-PAS-positive area to the epithelial area. (**D**–**G**) Colonic gene expression levels of TJ genes *Zo-1*, *Occludin*, *Claudin-1*, and *Claudin-5*. * *p* < 0.05 and ** *p* < 0.01.

**Figure 3:**
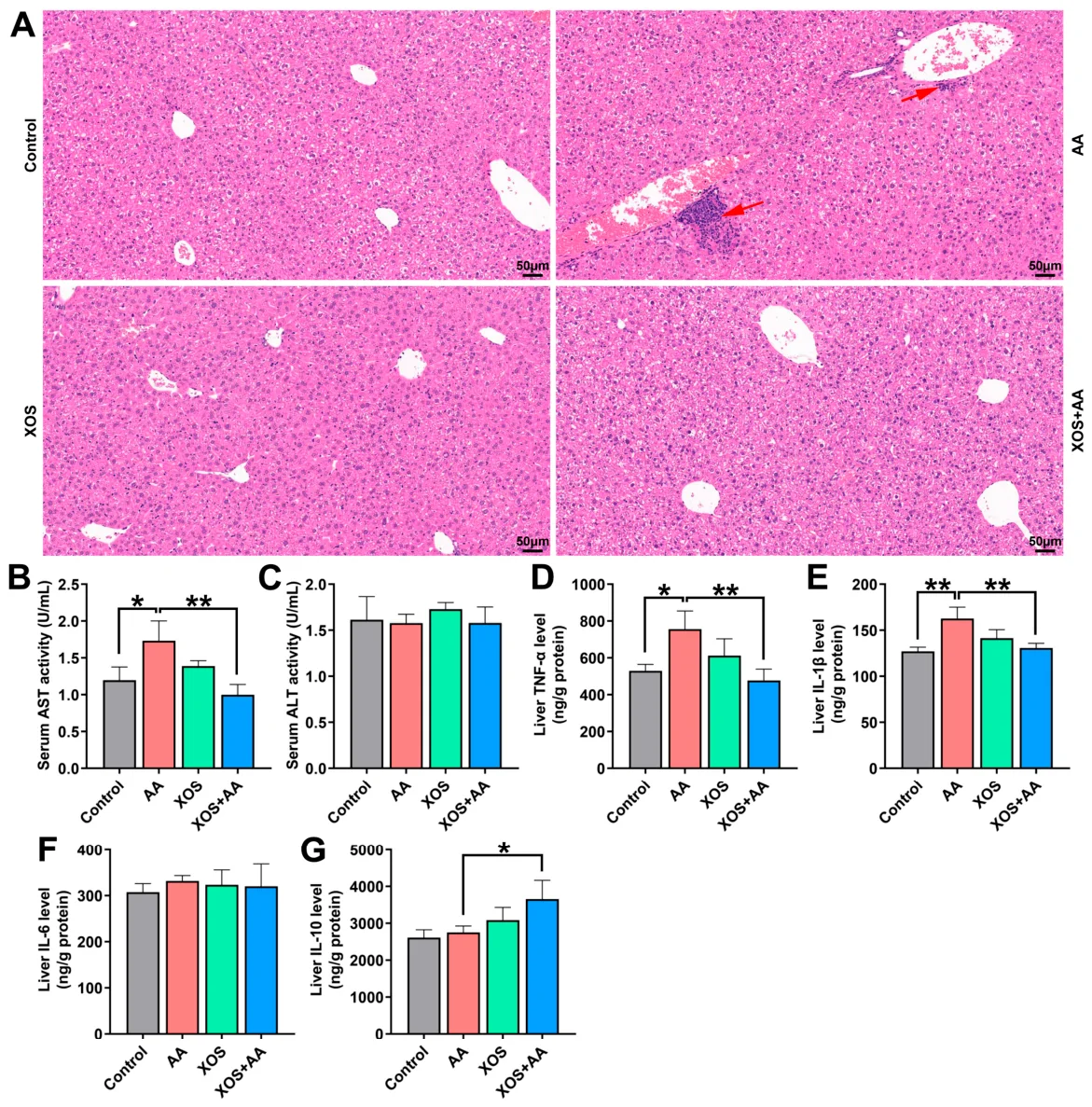
Dietary XOS mitigates AA-induced liver inflammation in mice (n = 6/group). (**A**) Representative H&E-stained liver tissue sections (scale bar: 50 μm). Red arrows indicate focal infiltration of lymphocytes and granulocytes. (**B**,**C**) Serum AST and ALT activities. (**D**–**G**) Hepatic levels of TNF-α, IL1-β, IL-6, and IL-10. * *p* < 0.05 and ** *p* < 0.01.

**Figure 4:**
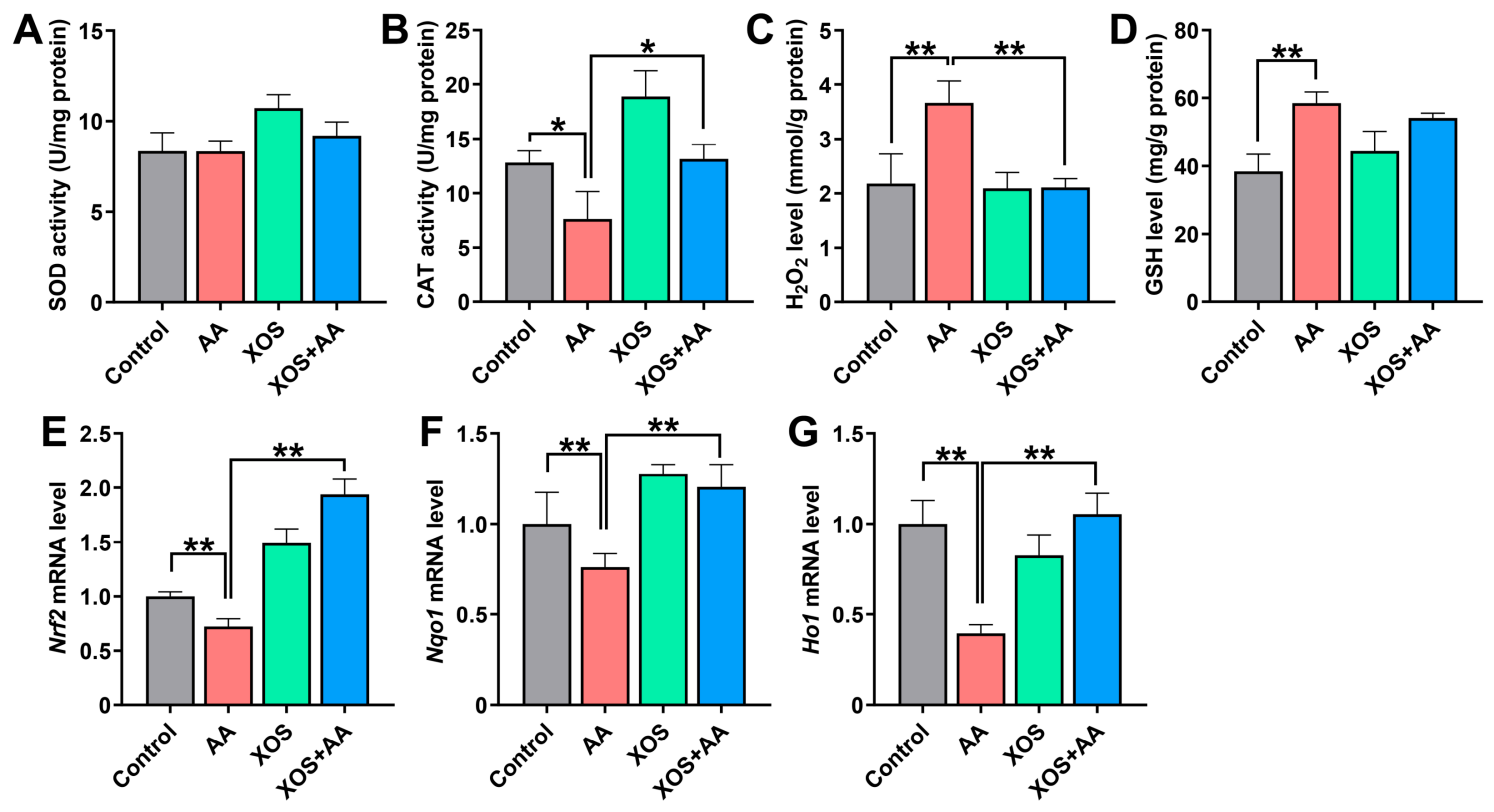
Dietary XOS mitigates AA-induced liver oxidative stress in mice (n = 6/group). (**A**,**B**) SOD and CAT activities. (**C**,**D**) H_2_O_2_ and GSH levels. (**E**–**G**) mRNA levels of *Nrf2*, *Nqo1*, and *Ho1*. * *p* < 0.05 and ** *p* < 0.01.

**Figure 5:**
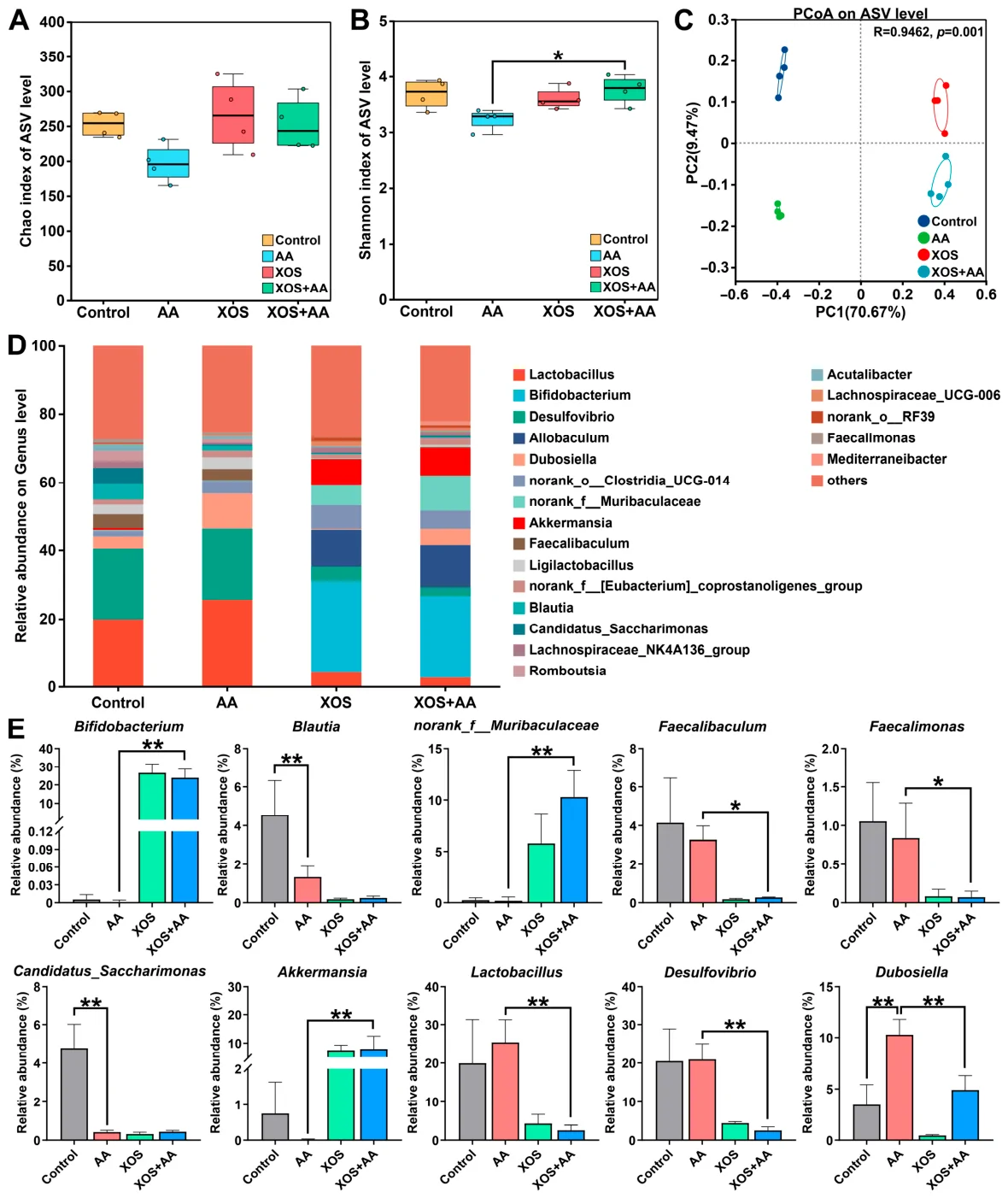
Dietary XOS positively modulates the cecal microbiota in mice exposed to AA (n = 4/group). (**A**,**B**) α-diversity indices. (**C**) Principal coordinate analysis (PCoA) plot based on Bray–Curtis distances. Differences in gut microbial community structure among groups were tested by ANOSIM, with 999 permutations. (**D**) Genus-level microbial composition. Each color represents a genus. (**E**) Genera showing significant differences (relative abundance ≥ 1.0% in at least one group). * *p* < 0.05 and ** *p* < 0.01.

**Figure 6:**
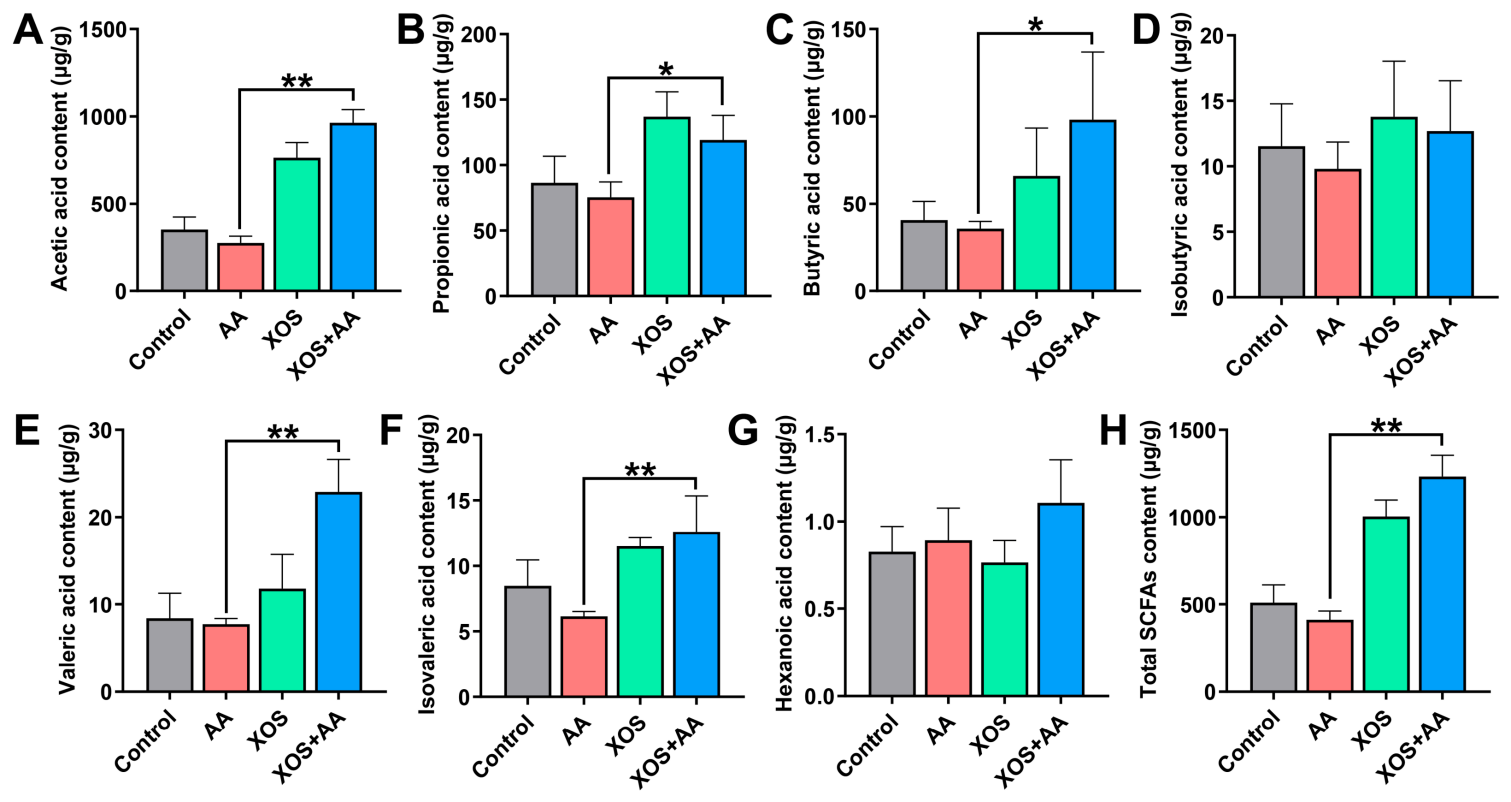
Dietary XOS elevates SCFA contents in the colonic contents of mice exposed to AA (n = 6/group). The contents of (**A**) Acetic, (**B**) propionic, (**C**) butyric, (**D**) isobutyric, (**E**) valeric, (**F**) isovaleric, and (**G**) hexanoic acid. (**H**) Total SCFA content. * *p* < 0.05, ** *p* < 0.01.

**Figure 7:**
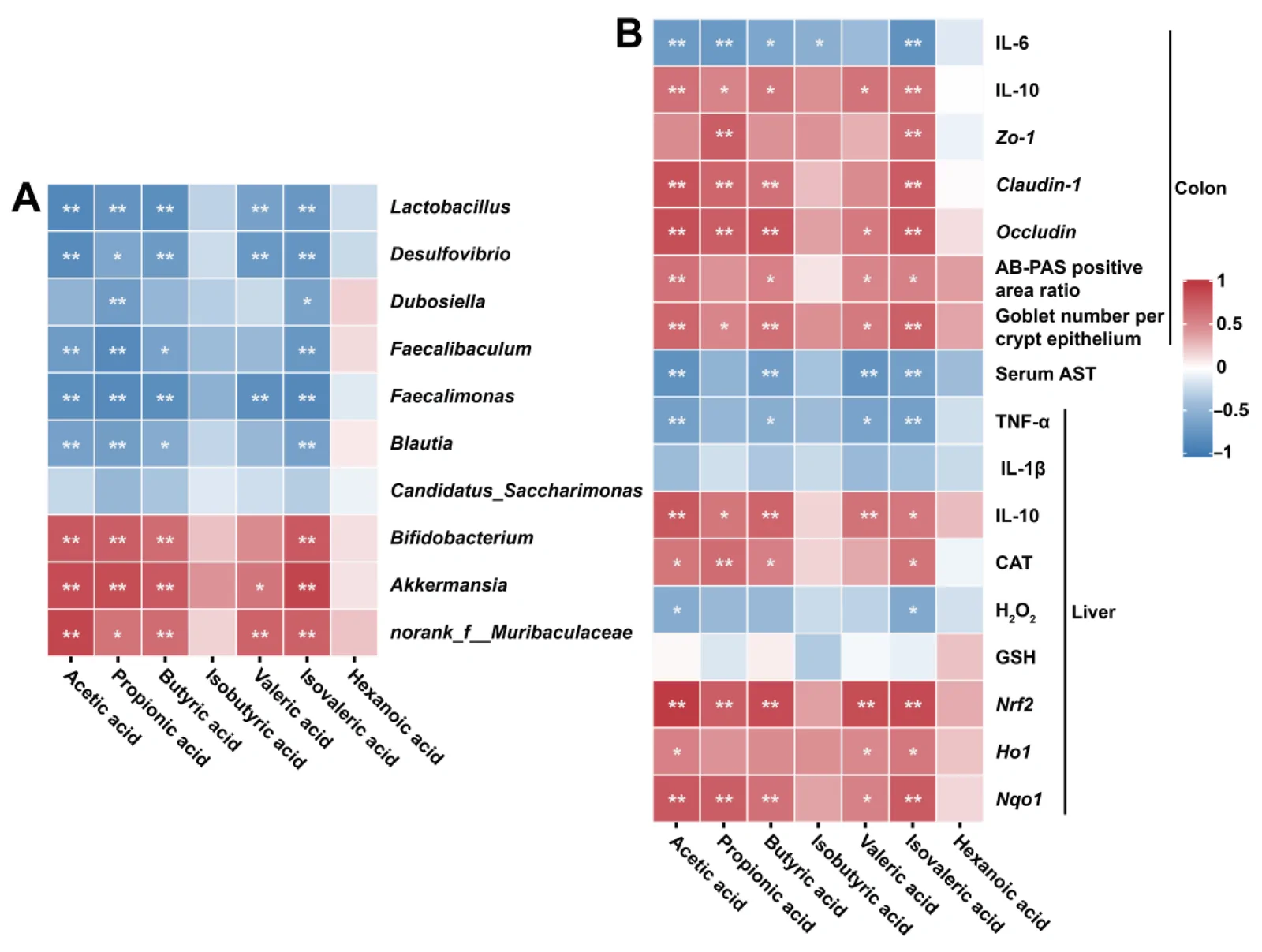
Spearman’s correlation analysis. (**A**) Correlations between differentially abundant genera and SCFAs; (**B**) Correlations between SCFAs and intestinal–liver indicators. * *p* < 0.05, ** *p* < 0.01.

**Table 1:** The physicochemical properties of XOS in this study.

Parameter	Content
XOS DP2–4 content (%, dry basis)	≥65.0
Moisture (%)	≤5.0
Ash (%)	≤0.3
pH	3.5–6.0
Total arsenic (as As) (mg/kg)	≤0.3%
Lead (as Pb) (mg/kg)	≤0.4%
Total viable count (CFU/g)	n = 5, c = 2, m = 100, M = 1000
Coliforms (MPN/g)	≤0.3
Yeasts (CFU/g)	≤25
Molds (CFU/g)	≤25
Pathogens	Not detected

## Data Availability

The raw data of 16S sequencing reported in this study have been uploaded to the NCBI Sequence Read Archive database (accession number: PRJNA1478807). The data are publicly available at https://www.ncbi.nlm.nih.gov/bioproject/PRJNA1478807, accessed on 6 June 2026.

## Supplementary Materials

The following supporting information can be downloaded at: https://www.mdpi.com/article/10.3390/biology15191684/s1, Table S1: Diet composition in this study; Table S2: The primer sequences for qPCR assay; Table S3: Sample-to-group mapping and cage information for the 16S rRNA sequencing samples; Table S4: Genus-level microbial composition; Table S5: Differentially abundant genera with a relative abundance ≥ 1.0% in at least one group. The References in the Supplementary Materials are cited in [70,71,72,73,74].
