# Supplementary material for: Corncob-Derived Xylo-Oligosaccharides Attenuate Acrylamide-Induced Gut–Liver Toxicity in Mice, Associated with Modulation of Gut Microbiota and SCFA Metabolism
Source: Biology (Basel). 2026 Sep 22;15(19):1684. doi: 10.3390/biology15191684 (PMC13663374; doi:10.3390/biology15191684)
Supplement: Supplementary file 1 [file biology-15-01684-s001.zip › biology-4562194-supplementary.pdf]

Supplementary Materials

Supplementary Methods

1. Bioinformatics analysis of 16S sequencing

For the raw sequencing reads, quality control filtering was first performed using Fastp, followed by merging of paired-end reads with FLASH. Denoising was carried out using the DADA2 plugin within Qiime2 to produce a bacterial amplicon sequence variant (ASV) abundance table. Taxonomic annotation of ASVs was then conducted via the Qiime2 platform against the SILVA 16S rRNA database. Based on the ASV data, alpha diversity was calculated with Mothur, while beta diversity analysis was performed using the R package *vegan*.

2. GC-MS/MS analysis

Specifically, GC conditions were as follows: separation was performed on an Agilent DB-FFAP capillary column (30 m × 250 μm × 0.25 μm). The oven temperature program started at 90 °C, was ramped at 10 °C/min to 160 °C, then further increased at 40 °C/min to 240 °C, and held for 5 min. Helium served as the carrier gas at a constant flow rate of 1.0 mL/min. MS conditions were set as follows: electron impact ionization (EI) mode at 70 eV; inlet temperature, 250 °C; ion source temperature, 230 °C; transfer line temperature, 250 °C; and quadrupole temperature, 150 °C.

Supplementary tables

Table S1 Diet composition in this study.

Ingredient	AIN93-M	AIN-93M containing 7.0% XOS
Casein (g)	140	140
L-Cystine (g)	1.80	1.80
Corn starch (g)	495.69	457.39
Maltodextrin (g)	125.00	125.00
Sucrose (g)	100.00	100.00
Cellulose (g)	50.00	50.00
Soybean oil (g)	40.00	40.00
Mineral mix (g)	35.00	35.00
Vitamin mix (g)	10.00	10.00
Choline bitartrate (g)	2.50	2.50
tert-Butylhydroquinone (g)	0.01	0.01
XOS-95P (g)	0	76.6
Red lake (g)	0	0.05
Total (g)	1000	1038.35
Carbohydrate (%)	73.1	74.1
Protein (%)	14.2	13.7
Fat (%)	4	3.9
Total (kcal)	3850	3850

Table S2 The primer sequences for qPCR assay

Gene name	Primer sequence (5’–3’)	Reference
Zo-1	F: TTCCCAGCTTATGAAAGGGTT<br>R: TCGCTTCTTTCAGGGCACCGTA	[70]
Claudin-1	F: GCCATCTACGAGGGACTG<br>R: GAGCAGGAAAGTAGGACACC	[70]
Claudin-5	F: ACTGCCTTCCTGGACCACAAC<br>R: CGCCAGCACAGATTCATACACCT	[70]
Occludin	F: TGCTTCATCGCTTCCTTAGTAA<br>R: GGGTTCACTCCCATTATGTACA	[71]
β-actin	F: GGTTGTCTCCTGCGACTTCA<br>R: TGGTCCAGGTTTCTTACTCC	[72]
Nrf2	F: CGAGATATACGCAGGAGAGGTAAGA<br>R: GCTCGACAATGTTCTCCAGCTT	[73]
Nqo1	F: AGCGTTCGGTATTACGATCC<br>R: AGTACAATCAGGGCTCTTCTCG	[74]
Ho1	F: AGGCTAAGACCGCCTTCCT<br>R: TGTGTTCCTCTGTCAGCATCA	[74]

Table S3 Sample-to-group mapping and cage information for the 16S rRNA sequencing samples

Sample ID	Mouse ID	Cage ID	Treatment
Contro_1	M1	C1	Control
Contro_2	M2	C1	Control
Contro_3	M3	C2	Control
Contro_4	M4	C2	Control
ACR_1	M5	C3	AA
ACR_2	M6	C3	AA
ACR_3	M7	C4	AA
ACR_4	M8	C4	AA
XOS_1	M9	C5	XOS
XOS_2	M10	C5	XOS
XOS_3	M11	C6	XOS
XOS_4	M12	C6	XOS
XOS+ACR_1	M13	C7	XOS+AA
XOS+ACR_2	M14	C7	XOS+AA
XOS+ACR_3	M15	C8	XOS+AA
XOS+ACR_4	M16	C8	XOS+AA

Table S4 Genus-level microbial composition

Genus	Control	AA	XOS	XOS+AA
Lactobacillus	19.89%	25.47%	4.34%	2.60%
Bifidobacterium	0.01%	0.00%	26.52%	23.89%
Desulfovibrio	20.48%	20.91%	4.42%	2.53%
Allobaculum	0.00%	0.00%	10.51%	12.41%
Dubosiella	3.49%	10.30%	0.47%	4.89%
norank_o__Clostridia_UCG-014	1.64%	3.72%	7.02%	5.43%
norank_f__Muribaculaceae	0.25%	0.20%	5.82%	10.26%
Akkermansia	0.75%	0.01%	7.52%	7.97%
Faecalibaculum	4.15%	3.30%	0.18%	0.27%
Ligilactobacillus	2.76%	3.33%	0.20%	0.59%
norank_f__[Eubacterium]_coprostanoligenes_group	1.51%	2.01%	1.12%	2.04%
Blautia	4.55%	1.31%	0.18%	0.25%
Candidatus_Saccharimonas	4.77%	0.42%	0.32%	0.45%
Lachnospiraceae_NK4A136_group	1.93%	0.60%	1.46%	1.12%
Romboutsia	2.96%	0.99%	0.00%	0.00%
Acutalibacter	1.86%	0.79%	0.38%	0.33%
Lachnospiraceae_UCG-006	0.15%	0.44%	1.45%	0.96%
norank_o__RF39	0.36%	0.06%	1.10%	0.72%
Faecalimonas	1.05%	0.83%	0.08%	0.07%
Mediterraneibacter	0.00%	0.00%	0.00%	1.03%
others	27.46%	25.32%	26.89%	22.19%

Table S5 Differentially abundant genera with a relative abundance ≥ 1.0% in at least one group

**Genus**	**Relative abundance (%)**			
	Control (Mean±SD)	AA (Mean±SD)	XOS (Mean±SD)	XOS+AA (Mean±SD)
Bifidobacterium	0.00541±0.008	0.00147±0.003	26.51±4.953	23.86±4.756
Blautia	4.56±1.762	1.33±0.574	0.1829±0.051	0.2482±0.102
norank_f__Muribaculaceae	0.2521±0.228	0.1984±0.366	5.797±2.876	10.29±2.529
Faecalibaculum	4.142±2.315	3.275±0.705	0.1827±0.0429	0.2782±0.016
Faecalimonas	1.054±0.501	0.8399±0.454	0.08411±0.092	0.0722±0.078
Candidatus_Saccharimonas	4.79±1.225	0.4211±0.097	0.3206±0.097	0.4436±0.064
Akkermansia	0.7469±0.888	0.01176±0.024	7.546±1.736	7.982±4.511
Lactobacillus	19.95±11.36	25.45±5.824	4.333±2.34	2.589±1.339
Desulfovibrio	20.53±8.341	20.94±4.121	4.416±0.372	2.534±0.948
Dubosiella	3.472±1.946	10.29±1.474	0.4703±0.069	4.918±1.365
